# COVID-19 Pandemic and Mental Health: Prevalence and Correlates of New-Onset Obsessive-Compulsive Symptoms in a Canadian Province

**DOI:** 10.3390/ijerph17196986

**Published:** 2020-09-24

**Authors:** Adam Abba-Aji, Daniel Li, Marianne Hrabok, Reham Shalaby, April Gusnowski, Wesley Vuong, Shireen Surood, Nnamdi Nkire, Xin-Min Li, Andrew J. Greenshaw, Vincent I.O. Agyapong

**Affiliations:** 1 Department of Psychiatry, Faculty of Medicine and Dentistry, University of Alberta, Edmonton, AB T6G 2B7, Canada; abbaaji@ualberta.ca (A.A.-A.); Daniel.Li@albertahealthservices.ca (D.L.); marianne.hrabok1@ucalgary.ca (M.H.); rshalaby@ualberta.ca (R.S.); Nnamdi.Nkire@albertahealthservices.ca (N.N.); Xinmin@ualberta.ca (X.-M.L.); andy.greenshaw@ualberta.ca (A.J.G.); 2 Addiction and Mental Health, Alberta Health Services, Edmonton, AB T5K 2J5, Canada; April.Gusnowski@albertahealthservices.ca (A.G.); Wesley.Vuong@albertahealthservices.ca (W.V.); shireen.surood@albertahealthservices.ca (S.S.); 3 Cumming School of Medicine, University of Calgary, Calgary, AB T2N 4Z6, Canada; 4 APEC Digital Hub for Mental Health, University of British Columbia, Vancouver, BC V6T 1Z4, Canada

**Keywords:** COVID-19, obsessive-compulsive disorder, stress, anxiety, generalized anxiety disorder, depression, major depressive disorder, public health, text, technology, pandemic

## Abstract

*Background*: This cross-sectional online survey investigates the prevalence of obsessive-compulsive disorder (OCD) symptoms at an early stage of the COVID-19 pandemic in Canada. *Methods*: OCD symptoms, moderate/high stress, likely generalized anxiety disorder (GAD) and likely major depressive disorder (MDD) were assessed with the Brief Obsessive-Compulsive Scale (BOCS), Perceived Stress Scale (PSS), Generalized Anxiety Disorder 7-item (GAD-7) scale, and Patient Health Questionnaire-9 (PHQ-9) scale, respectively. *Results*: Out of 32,805 individuals subscribed to Text4Hope, 6041 completed an online survey; the response rate was 18.4%. Overall, 60.3% of respondents reported onset of OCD symptoms and 53.8% had compulsions to wash hands during the COVID-19 pandemic. Respondents who showed OCD symptoms only since the start of COVID-19 were significantly more likely to have moderate/high stress (z = 6.4, *p* < 0.001), likely GAD (z = 6.0, *p* < 0.001), and likely MDD (z = 2.7, *p* < 0.01). Similarly, respondents who engaged in compulsive hand washing were significantly more likely to have moderate/high stress (z = 4.6, *p* < 0.001) and likely GAD (z = 4.6 *p* < 0.001), but not likely MDD (z = 1.4, *p* = 0.16). *Conclusion*: The prevalence of OCD symptoms increased during the COVID-19 pandemic, at a rate significantly higher than pre-pandemic rates reported for the sample population. Presenting with OCD symptoms increased the likelihood of presenting with elevated stress, likely GAD, and likely MDD.

## 1. Introduction

The 2019 coronavirus disease (COVID-19) can cause a form of severe acute respiratory syndrome that may rapidly lead to death in vulnerable persons. It has a high droplet transmission rate from person to person, with a fatality rate of 2–5% [1,2]. In March of 2020, approximately 136 countries imposed stringent measures to limit the spread of COVID-19, including staying at home, physical distancing of 2 m, and prohibition of social gatherings. This has been accompanied by extensive public health campaigns on regular hand washing, hygiene, and personal protective equipment (PPE) such as face masks and gloves.

While these measures are important, they may negatively impact the mental health of vulnerable individuals. Limitations and restrictions imposed on individuals aimed towards the protection of the public from communicable diseases can result in mental illness [3]. In this context, public perception is positively correlated with the psychological impact of an outbreak [4]. An important risk factor for mental illness during a pandemic is an individual’s constant worry about self and family members [4]. Excessive worry is an accepted etiologic factor in the development of obsessive-compulsive disorder (OCD) symptoms [5].

OCD is characterized by obsessions, including fear of contamination by dirt or germs, which generate distress that frequently results in compulsions to temporarily alleviate anxiety. While the lifetime prevalence of OCD symptoms is over 25% [6], the lifetime prevalence of the full disorder is much less, estimated at 2–3% for the general population [7]. OCD is highly comorbid with anxiety disorders and depression [8], including major depressive disorder (MDD), social anxiety disorder and generalized anxiety disorder (GAD) [9]. Individuals with OCD may experience a significant impairment in psychosocial and occupational level of functioning, leading to, or exacerbating, poor quality of life [10]. In the absence of early intervention, OCD can run a chronic course [11]. The diminished quality of life seen in people diagnosed with OCD is comparable to the level observed with other severe mental disorders like schizophrenia [12]. The etiology of OCD is associated with the interplay of multiple risk factors, such as gene, environment and life stressors [13].

There is a paucity of data describing the prevalence of OCD symptoms during communicable disease pandemics, despite the fact that these represent a period of time when people are required to be hypervigilant about preventing the threat of the contamination of self and others. Our study aimed to increase our knowledge in this area by investigating associations between OCD symptoms and symptoms of perceived stress, GAD and MDD using a population-based, cross-sectional survey design during the COVID-19 pandemic. Stressful life events may precipitate or predispose individuals to development of OCD symptoms. The intense focus on danger of contamination from a virus during COVID-19, with the ensuing major disruption of personal health, social routines, health-systems and the economy, may increase the risks associated with the genesis of OCD symptoms in the population [14].

Data collection for this study occurred during the initial phase of the COVID-19 epidemic in the province of Alberta, Canada, currently comprising a census population of 4,413,146 persons [15]. At the close of the survey collection on 30 March 2020, 690 COVID-19 cases were identified in the province, of which 65 were suspected to be community-acquired, 94 were recovered, and 47 had been hospitalized, with a total of 17 admissions to intensive care units [16]. The objectives of our study are to determine the prevalence and correlates of OCD symptoms amongst a cross-section of Canadian subscribers to the Text4Hope program during the COVID-19 pandemic, and to examine the association between new onset OCD symptoms and high/moderate perceived stress, likely GAD, and likely MDD.

## 2. Materials and Methods

This is a cross-sectional study based on data collected online from subscribers to Text4Hope, a daily supportive text message service launched in partnership with Alberta Health Services, the Provincial health authority to support the mental health of Alberta residents. Individuals self-subscribed to Text4Hope by texting “COVID19HOPE” to a designated short code number. Subscribers receive a link to the online survey designed to gather demographic variables such as age, gender, ethnicity, education, employment status, relationship status and housing status. The 10 min (average duration) survey also assessed obsessive-compulsive symptoms with two items on the Brief Obsessive-Compulsive Scale (BOCS) [17], perceived stress with the Perceived Stress Scale (PSS) [18], likely GAD with the Generalized Anxiety Disorder 7-item (GAD-7) scale [19], and likely MDD with the Patient Health Questionnaire-9 (PHQ-9) scale [20]. The two modified questions from the BOCS were:

- I am worried about dirt, germs and viruses. Ex. Fear of getting germs from touching door handles or shaking hands or sitting in certain chairs or seats or fear of getting COVID-19.
- I wash my hands very often or in a special way to be sure I am not dirty or contaminated.

Ex. Washing one’s hands many times a day or for long periods after touching, or thinking one has touched, a contaminated object.

The responses to the above questions were modified to these three Likert scales: “Only during COVID-19 Pandemic”, “Before and During COVID-19 Pandemic,” or “Never”.

The study was approved by the University of Alberta Human Ethics Review Board (Pro00086163), and consent was implied if the participants completed the online survey and submitted responses. With an estimated population of 4,371,316 in Alberta, we calculated the minimum sample size required to estimate mental disorder prevalence rates with a confidence level of 99% and a 2% margin of error as *n* = 4200. Given the expected response rate of 20% [21], we planned to extract data after at least 20,785 individuals had subscribed to Text4Hope. Data were collected 23–30 March 2020, with 32,805 subscriptions to Text4Hope, thus exceeding the target sample size. The data were analyzed with Statistical Package for Social Sciences (SPSS) version 20 [22] using descriptive statistics and Chi-Square tests. Two tailed significance (*p* < 0.05) was used to assess the relationship between obsessive-compulsive symptoms and other mental health variables. For mental health variables with statistically significant relationship with OCD symptoms, we performed post-hoc analysis, comparing those who had new onset OCD symptoms with those who had never had OCD symptoms and reported corresponding z-scores, adjusted residuals and *p*-values. Given the cross-sectional study design, there was no imputation for missing data and the results were based on completed survey responses.

## 3. Results

Of the 32,805 individuals invited to complete an online survey, 6041 responded, yielding a response rate of 18.4%. Table 1 provides descriptive summaries of the demographic and clinical characteristics of the respondents.

The data displayed in Table 1 indicate that 60.3% of respondents had obsessions related to contamination with dirt, germs or viruses, and 53.8% had compulsions to wash hands repeatedly or in a special way, which both started during the COVID-19 pandemic. The one-week prevalence rates for moderate/high stress, likely GAD and likely MDD in Alberta were 84.9%, 46.7% and 41.4%, respectively.

Table 2 suggests there were statistically significant correlations between obsessions related to dirt, germs and viruses, and all demographic variables assessed. The groups of respondents who identified as male, over 60 years of age, Caucasian, with post-secondary education, retired, widowed, or living in their own homes contained a higher proportion of respondents who expressed worry related to contamination with dirt, germs and viruses, compared to other respondents.

Table 3 suggests that all demographic variables except gender and relationship status had statistically significant relationships with compulsive hand washing. Groups of respondents who identified as being over 60 years of age, Caucasian, with post-secondary education, retired or home owners had higher proportions of respondents who were engaged in compulsive hand washing compared to other respondents.

The data displayed in Table 4 indicate significant correlations between obsessions about dirt, germs and viruses, and between those who engaged in compulsive hand washing and the likelihood that respondents had moderate/high stress, likely GAD and likely MDD. Post-hoc analysis using adjusted residuals indicates that respondents who were worried about dirt, germs and viruses only since the start of the COVID-19 pandemic were significantly more likely to have moderate/high stress (*z* = 6.4, *p* < 0.001), likely GAD (*z* = 6.0, *p* < 0.001) and likely MDD (*z* = 2.7, *p* < 0.01) compared to respondents who have never been worried about dirt, germs and viruses. Similarly, respondents who engage in compulsive hand washing were significantly more likely to have moderate/high stress (*z* = 4.6, *p* < 0.001) and likely GAD (*z* = 4.6 *p* < 0.001), but not likely MDD (*z* = 1.4, *p* = 0.16), compared to respondents who have never engaged in compulsive hand washing.

## 4. Discussion

To our knowledge, this population-based, cross-sectional survey of 6501 respondents during the COVID-19 pandemic is the first to report the prevalence of OCD symptoms and their correlation with stress, anxiety and depression symptoms. The high levels of stress, anxiety and depression symptoms underscore the need for focused the mental health prevention, intervention and follow-up of affected vulnerable groups during the COVID-19 pandemic. These results align with the prevalence rates in a survey-based measurement of 1257 health care workers in fever clinics and wards for COVID-19 patients in China, which also used the PHQ-9 and GAD-7, in which participants reported high rates of distress (71.5%), anxiety (44.6%) and depression (50.4%) [23]. Similarly, in another survey study of the initial stage of the COVID-19 epidemic in China, 1652 respondents rated the psychological impact as moderate-to-severe, with one-third reporting moderate-to-severe anxiety, and 16.5% reporting moderate-to-severe depressive symptoms [24]. Further, an Italian study by Magnavita and colleagues [25] found similar levels of anxiety and depression during a comparable period in the pandemic. These studies both show similar findings in different geographical jurisdictions. The results of this early-stage pandemic study support the proposal that surveying the OCD symptom dimensions are important for future pandemic planning, where strict public health measures (e.g., requiring regular handwashing, use of facemask and social distancing) are implemented or enforced. While 25.1–33.1% of the sample reported pre-COVID-19 OCD symptoms, an additional 60.3% were obsessed with fears of contamination and 53.8% had compulsive hand-washing. Post-hoc analysis revealed that those with new onset OCD symptoms are statistically more likely to have high stress, likely GAD, and likely MDD. This is a 10 to 30-fold increase in OCD symptoms relative to the prevalence reported in the pre-pandemic general population [6].

These results indicate that both previous and new onset OCD contamination symptoms correlate with, and may serve as a marker for, a moderate/high stress group that is more vulnerable to GAD and MDD during COVID-19. Because global pandemics are associated with increased somatic and cognitive anxiety [22,26,27], the combination of this stress and specific OCD contamination worries may result in negative-valence cognitive ruminations that activate vulnerabilities to GAD and MDD. The correlation among OCD, GAD and MDD has been explained by the overlap of common genetics, neurobiology, and shared psychological constructs [28,29].

The lifetime prevalence of OCD symptoms is over 25%, but only a small proportion fulfill the full criteria for OCD, with a lifetime prevalence of 2.1% [6]. In a four year follow-up of a subgroup of 181 severe acute respiratory syndrome (SARS) survivors that used the Structured Clinical Interview for Diagnostic and Statistical Manual of Mental Disorders IV (DSM-IV), common diagnoses included post-traumatic stress disorder (54.5%), depression (39.0%) and OCD (15.6%)—the latter of which is seven times higher than the lifetime prevalence rate of OCD (2.1%) [30]. Thus, whether the new-onset OCD symptoms observed in our study are related to true OCD disease risk, are an expression of specific phobia-type risks in the context of COVID-19, or are a combination of both, will be for future research to determine. It may also be that the obsessive-compulsive symptoms are an adaptive response to protect the self and others from the virus, as the behaviors sampled are in line with public health recommendations. In order to evaluate the adaptive nature of OCD symptoms during the COVID-19 pandemic, the persistence or resolution of these symptoms must be determined in the recovery stage of the pandemic when the acute phase has ended.

In this study, OCD contamination symptoms were associated with male gender, an age over 60 years, Caucasian ethnicity, post-secondary education, retired employment status, widowed relationship status, and living in your own home. These findings are in contrast with other studies reporting significant association of OCD with younger age, marital status [31] and female gender, specifically with OCD contamination symptoms [32]. The mean age of respondents in our study is 42 years (age range 11–88 years), which is higher than the generally reported mean age of the onset of 17.9 years for OCD [33]. This is important because the onset of OCD prior to 20 years of age is associated with a poor prognosis, whereas an onset over 20 tends to have a shorter course and better outcomes [34]. Therefore, given the later age of onset of OCD symptoms in our study, those who develop OCD symptoms during the COVID-19 pandemic are likely to have a better prognosis.

This study suggests that OCD symptoms are associated with the liabilities of increased stress, GAD and MDD. In balance, in a survey of 705 Hong Kong and 1201 Singaporean residents during the SARS epidemic, general anxiety measured using the State-Trait Anxiety Inventory (STAI) was adaptive and positively associated with the adoption of personal protection measures in Hong Kong [35]. Determining to what degree GAD and OCD symptoms are adaptive versus a liability during the initial phase of the COVID-19 pandemic will require further work. This study, however, adds the association of depressive symptoms in a pandemic to obsessive symptoms, which may indicate a further risk of vulnerability to adverse psychological sequelae.

The limitations of the present study include the use of a self-reported questionnaire for cognitive and behavioral symptoms of OCD, GAD and MDD that would require objective clinical assessment for definitive diagnosis. Secondly, our study is not representative of the population in Alberta either by age or gender [36], and so our findings may not be generalized to the entire population. Thirdly, we cannot claim to have sufficient statistical power to clearly determine the strength of the correlation between the COVID-19 pandemic and the onset of OCD. In addition, the associated demographic and clinical factors as determined by Chi-Square test could have confounding factors, which means the effect size estimates could be overestimated. To minimize the influence of potential confounding factors, the research team plan to use machine learning methods to develop models on a bigger data set, as described in the study protocol. Furthermore, the increased OCD symptoms may be a reflection of the real threat posed by COVID-19. As a result, it is possible that once the pandemic is over, a proportion of those with new-onset OCD symptoms would not continue to report these symptoms. Post-pandemic studies are therefore required to determine and understand the temporal relationship between OCD symptoms and the COVID-19 pandemic. We used well-validated and standardized scales to mitigate the risk of information bias that could possibly be introduced in a self-reported questionnaire. However, the lack of randomization may have introduced selection bias and, therefore, effected the strength of the generalizability of our finding. Lastly, this survey is unable to measure the direct effect of COVID-19 on persons with a confirmed diagnosis of OCD, and this is an interesting area for future investigation. Our data support the proposal that public health advice during pandemics should incorporate mental health wellness campaigns aiming to reduce the psychological impact of pandemics. There is increasing attention being paid to this need in the media, and our data may serve to provide evidence-based support for such policy implementation.

## 5. Conclusions

The results of our study reveal a surge in reported obsessive-compulsive symptoms with corresponding high level of stress, likely GAD and likely MDD during the COVID-19 pandemic. The use of a large population-based sample of Canadians is a significant strength of this study. As our findings correspond to some prevalence rates observed in recent studies from different geographic jurisdictions [23,24], as described above, conclusions drawn from our data regarding the prevalence of OCD symptoms, likely GAD and likely MDD correlates are likely fairly representative of the general Canadian population. Innovative and cost-effective interventions with the capability to be deployed quickly at the population level, such as supportive text messaging which is free to the end user, does not require expensive data plans, can reach thousands of people simultaneously, is independent of geographic location [21,37,38,39,40,41,42,43], and could be particularly useful for those experiencing OCD symptoms and those who are at a higher risk of experiencing stress, anxiety and depression during the COVID-19 pandemic.

## Figures and Tables

**Table 1:** Demographic and clinical characteristics of respondents.

Variables	Overall
Gender	
Male	740 (12.4%)
Female	5185 (86.6%)
Other Gender	61 (1.0%)
Age (Years)	
≤25	640 (10.9%)
26–40	2174 (37%)
41–60	2539 (43.3%)
>60	517 (8.8%)
Ethnicity	
Caucasian	4910 (82.2%)
Indigenous	205 (3.4%)
Asian	301 (5.0%)
Other	554 (9.3%)
Education	
Less than High School Diploma	218 (3.6%)
High School Diploma	583 (9.7%)
Post-Secondary Education	5123 (85.6%)
Other Education	59 (1.0%)
Employment status	
Employed	3726 (72.1%)
Unemployed	719 (13.9%)
Retired	399 (7.7%)
Student	322 (6.2%)
Relationship status	
Married/Common-law/Partnered	4284 (71.6%)
Separated/Divorced	438 (7.3%)
Widowed	93 (1.6%)
Single	1105 (18.5%)
Other	62 (1.0%)
Housing status	
Own Home	3917 (66.6%)
Living with Family	548 (9.3%)
Renting	1355 (23.0%)
Other	63 (1.1%)
Worried about dirt, germs, and viruses	
Only since COVID-19 pandemic	3111 (60.3%)
Before and during COVID-19 pandemic	1293 (25.1%)
Never	753 (14.6%)
Wash hands very often or in a special way to be sure he/she is not dirty or contaminated	
Only since COVID-19 pandemic	2771 (53.8%)
Before and during COVID-19 pandemic	1702 (33.0%)
Never	678 (13.2%)
Respondents had moderate/high stress ^a^	4689 (84.9%)
Respondents had likely GAD ^b^	2362 (46.7%)
Respondents had likely MDD ^c^	2130 (41.3%)

^a^ Moderate or high stress defined as Perceived Stress Scale score ≥14. ^b^ Likely GAD defined as Generalized Anxiety Disorder-7 scale score ≥10. ^c^ Likely MDD defined as Patient Health Questionnaire -9 scale score ≥10.

**Table 2:** Demographic characteristics of respondents with obsessive symptoms (dirt, germs, and viruses).

Variables	Worried about Dirt, Germs, and Viruses			*p*-Value	* Effect Size (Phi)	
	Only Since COVID-19 Pandemic “After”	Before and During COVID-19 Pandemic	Never			
Gender						
Male	388 (63.2%)	106 (17.3%)	120 (19.5%)	<0.001	0.08	
Female	2694 (60.1%)	1168 (26%)	623 (13.9%)			
Other Gender	25 (52.1%)	16 (33.3%)	7 (14.6%)			
Age (Years)						
≤25	308 (55.7%)	166 (30%)	79 (14.3%)	<0.001	0.06	
26–40	1146 (60.8%)	494 (26.2%)	246 (13%)			
41–60	1333 (60.9%)	513 (23.4%)	344 (15.7%)			
>60	281 (62.0%)	96 (21.2%)	76 (16.8%)			
Ethnicity						
Caucasian	2630 (61.2%)	1023 (23.8%)	642 (14.9%)	<0.001	0.07	
Indigenous	101 (56.4%)	57 (31.8%)	21 (11.7%)			
Asian	131 (58.5%)	73 (32.6%)	20 (8.9%)			
Other	241 (54.8%)	131 (29.8%)	68 (15.5%)			
Education						
Less than High School Diploma	93 (52.8%)	53 (30.1%)	30 (17.0%)	0.01	0.06	
High School Diploma	280 (57.7%)	133 (27.4%)	72 (14.8%)			
Post-Secondary Education	2714 (61.0%)	1085 (24.4%)	647 (14.6%)			
Other Education	19 (45.2%)	19 (45.2%)	4 (9.5%)			
Employment status						
Employed	1994 (62.4%)	739 (23.1%)	460 (14.4%)	<0.01	0.07	
Unemployed	327 (54.0%)	186 (30.7%)	92 (15.2%)			
Retired	225 (63.2%)	75 (21.1%)	56 (15.7%)			
Student	172 (59.9%)	73 25.4(%)	42 (14.6%)			
Relationship status						
Married/Common-law/Partnered	2287 (61.6%)	911 (24.5%)	515 (13.9%)	<0.001	0.07	
Separated/Divorced	243 (62.5%)	87 (22.4%)	59 (15.2%)			
Widowed	56 (66.7%)	18 (21.4%)	10 (11.9%)			
Single	507 (55.0%)	257 (27.9%)	158 (17.1%)			
Other	16 (38.1%)	17 (40.5%)	9 (21.4%)			
Housing status						
Own Home	2087 (61.4%)	807 (23.7%)	505 (14.9%)	<0.001	0.07	
Living with Family	249 (52.1%)	142 (29.7%)	87 (18.2%)			
Renting	722 (60.7%)	320 (26.9%)	148 (12.4%)			
Other	26 (56.5%)	16 (34.8%)	4 (8.7%)			

* Effect size as measured by Phi: A value of 1 is considered a small effect, 3 a medium effect and 5 a large effect.

**Table 3:** Demographic characteristics of respondents with compulsive symptoms (repeated hand washing).

Variables	Wash Hands Very Often or in a Special Way to Be Sure He/She Is Not Dirty or Contaminated			*p*-Value	* Effect Size (Phi)
	Only since COVID-19 Pandemic “After”	Before and during COVID-19 Pandemic	Never		
Gender					
Male	350 (57.3%)	189 (30.9%)	72 (11.8%)	0.06	0.04
Female	2395 (53.4%)	1495 (33.4%)	592 (13.2%)		
Other Gender	22 (45.8%)	14 (29.2%)	12 (25.0%)		
Age (Years)					
≤25	264 (47.7%)	238 (43.0%)	51 (9.2%)	<0.001	0.11
26–40	989 (52.5%)	658 (34.9%)	237 (12.6%)		
41–60	1198 (54.7%)	656 (30.0%)	335 (15.3%)		
>60	284 (63.3%)	121 (26.9%)	44 (9.8%)		
Ethnicity					
Caucasian	2363 (55.1%)	1348 (31.4%)	577 (13.5%)	<0.001	0.08
Indigenous	82 (45.8%)	79 (44.1%)	18 (10.1%)		
Asian	119 (53.1%)	83 (37.1%)	22 (9.8%)		
Other	197 (44.7%)	184 (41.7%)	60 (13.6%)		
Education					
Less than High School Diploma	79 (44.6%)	83 (46.9%)	15 (8.5%)	<0.001	0.09
High School Diploma	251 (51.9%)	196 (40.5%)	37 (7.6%)		
Post-Secondary Education	2417 (54.4%)	1403 (31.6%)	620 (14.0%)		
Other Education	19 (45.2%)	17 (40.5%)	6 (14.3%)		
Employment status					
Employed	1789 (56.1%)	991 (31.1%)	408 (12.8%)	<0.001	0.09
Unemployed	276 (45.5%)	242 (39.9%)	89 (14.7%)		
Retired	220 (62.1%)	92 (26.0%)	42 (11.9%)		
Student	142 (49.5%)	112 (39.0%)	33 (11.5%)		
Relationship status					
Married/Common-law/Partnered	2030 (54.7%)	1182 (31.9%)	496 (13.4%)	0.09	0.05
Separated/Divorced	205 (52.6%)	126 (32.3%)	59 (15.1%)		
Widowed	45 (53.6%)	32 (38.1%)	7 (8.3%)		
Single	468 (50.8%)	342 (37.1%)	111 (12.1%)		
Other	21 (51.2%)	16 (39.0%)	4 (9.8%)		
Housing status					
Own Home	1894 (55.8%)	1022 (30.1%)	477 (14.1%)	<0.001	0.10
Living with Family	208 (43.5%)	215 (45.0%)	55 (11.5%)		
Renting	626 (52.6%)	430 (36.1%)	134 (11.3%)		
Other	19 (41.3%)	20 (43.5%)	7 (15.2%)		

* Effect size as measured by Phi: A value of 1 is considered a small effect, 3 a medium effect, and 5 a large effect.

**Table 4:** Chi-Square test of association between obsessive-compulsive symptoms and perceived stress, likely GAD and likely MDD.

Variables	Perceived Stress			Generalized Anxiety Disorder			Major Depressive Disorder			
	Moderate/High Stress	*p*-Value	Effect Size (Phi)	GAD Likely	*p*-Value	Effect Size (Phi)	MDD Likely	*p*-Value	* Effect Size (Phi)	
Worried about dirt, germs, and viruses										
Only since COVID-19 pandemic	2656 (85.6%)	<0.001	0.11	1445 (47.3%)	<0.001	0.10	1276 (41.1%)	<0.001	0.06	
Before and during COVID-19 pandemic	1133 (88.0%)			656 (51.7%)			581 (45.1%)			
Never	571 (76.0%)			258 (35.1%)			268 (35.6%)			
Wash hands very often or in a special way to be sure hands are not dirty or contaminated										
Only since COVID-19 pandemic	2359 (85.3%)	<0.001	0.08	1288 (47.3%)	<0.001	0.07	1111 (40.1%)	0.01	0.05	
Before and during COVID-19 pandemic	1471 (86.8%)			820 (49.2%)			759 (44.8%)			
Never	526 (78.0%)			249 (37.4%)			252 (37.2%)			

* Effect size as measured by Phi: A value of 1 is considered a small effect, 3 a medium effect, and 5 a large effect.
